# Mediating role of intrinsic learning motivation in the relationship between future time perspective and classroom disengagement among Chinese nursing students: a cross-sectional study

**DOI:** 10.1186/s12912-025-04280-6

**Published:** 2026-01-08

**Authors:** Hongxia Zhao, Shijia Qin, Wenxiu Ding, Zijian Li, Rong Gao

**Affiliations:** 1 Xi’an Jiaotong University City College, Xi’an, 710000 China; 2 Xi’an Medical University, Xi’an, 710000 China

**Keywords:** Nursing students, Future time perspective, Intrinsic learning motivation, Classroom disengagement

## Abstract

**Background:**

Nursing students, as future frontline healthcare providers, operate in a complex and demanding field where maintaining academic engagement is crucial for effective learning and professional development. However, this engagement is often challenged by various factors. Although future time perspective (FTP) is known to influence student engagement, the mediating pathway through intrinsic learning motivation (ILM) remains underexplored in nursing education. This study is among the first to provide a large-scale empirical validation of this specific FTP–ILM–classroom disengagement pathway in the context of Chinese nursing education. It aims to clarify this mediating mechanism and provide practical implications for reducing disengagement and enhancing educational quality.

**Methods:**

This cross-sectional study used convenience sampling to investigate 889 nursing students from six universities in Shaanxi Province from December 2024 to March 2025. The instruments included the Future Time Perspective Scale, the Intrinsic Learning Motivation Scale, and the Classroom Disengagement Scale. Data were analyzed by using IBM SPSS Statistics 26.0 for descriptive statistics, correlation analysis, and mediation analysis.

**Results:**

A total of 920 nursing students completed the questionnaires, resulting in 889 valid responses (96.6% response rate). Invalid questionnaires were primarily due to incomplete data. The average score on the Classroom Disengagement Scale was 88.055 ± 12.085, with a range from 40 to 120. Pearson correlation analysis showed a positive relationship between FTP and ILM (*r* = 0.613, *p* < 0.01), and negative correlations between ILM and classroom disengagement (*r* = −0.738, *p* < 0.01), and between FTP and classroom disengagement (*r* = −0.743, *p* < 0.01). Mediation analysis using the Bootstrap method revealed a significant indirect effect of FTP on classroom disengagement through ILM (effect = −0.279, 95% CI: −0.319, −0.241).

**Conclusion:**

This study highlights the significant roles of FTP and ILM in classroom disengagement among nursing students, with ILM acting as a mediator. To mitigate classroom disengagement and improve nursing educational quality, nursing educators should implement strategies to cultivate a positive FTP and strengthen ILM among students.

**Clinical trial number:**

Not applicable.

**Supplementary Information:**

The online version contains supplementary material available at 10.1186/s12912-025-04280-6.

## Highlights

1. Future time perspective significantly reduces classroom disengagement among nursing students both directly and through intrinsic learning motivation.

2. Intrinsic learning motivation acts as a critical mediator, attenuating the negative impact of classroom disengagement by enhancing students’ connection to future goals.

3. Integrating future time perspective interventions and enhancing intrinsic learning motivation in nursing education can improve academic engagement and professional development, potentially reducing nursing brain drain and improving nursing quality.

## Introduction

In the contemporary higher education system, insufficient academic engagement has been identified as a major factor compromising educational quality [1]. Classroom disengagement, a salient manifestation of this inadequacy, describes a behavioral pattern in which students are physically present but mentally disengaged from the learning process. It is typically characterized by inattention, the passive completion of learning tasks, and superficial interaction with course materials. This phenomenon not only hinders students’ immediate academic progress but may also adversely affect their long-term professional development. A 2023 national survey highlighted the scale of this issue, revealing that 70.8% of students reported being mentally disengaged for more than half of their class time, whereas only 6.7% remained consistently attentive throughout lectures [2]. The use of social media is widely recognized as a prevalent source of distraction in the classroom [3]. In the context of nursing education, classroom disengagement is similarly common. A cross-sectional survey of 628 undergraduate nursing students from eight Chinese universities indicated moderate levels of this issue [4]. Nursing education presents unique challenges due to its highly practical nature and the significant responsibilities involved, such as intensive skill training, pressures from clinical practice, and the need to integrate extensive theoretical knowledge with hands-on application [5]. In such a demanding environment, classroom disengagement can have serious consequences. It may result in an inadequate mastery of clinical skills, which can subsequently impair clinical decision-making abilities and ultimately hinder the development of a strong professional identity [6].

Empirical research on classroom disengagement remains relatively limited within Western higher education systems. This may be attributable to factors such as flexible attendance policies and greater student autonomy in course selection [7, 8]. In contrast, Chinese universities typically implement strict attendance monitoring systems, a context in which passive participation has emerged as a common form of disengagement [9]. This distinction underscores the systemic and cultural differences shaping educational experiences. For nursing students, classroom disengagement can significantly hinder the acquisition of core competencies, reduce active learning, and adversely affect educational outcomes [5]. In clinical settings, this may manifest as a theory-practice gap, thereby elevating potential medical risks. Furthermore, sustained disengagement is likely to erode intrinsic learning motivation (ILM) and weaken the development of a professional identity, which may negatively influence future career choices and professional conduct [6]. The mechanisms underlying covert classroom disengagement have long been a focus of research in educational psychology. Current evidence has predominantly analyzed influencing factors through motivational frameworks, among which intrinsic learning motivation (ILM) has been identified as a critical protective factor [4, 10]. Empirical studies indicate that ILM represents a robust and sustainable form of motivation, playing a vital role in promoting active learning behaviors and fostering self-directed competency development [11, 12]. According to Self-Determination Theory (SDT), ILM reflects an individual’s interest and enjoyment in the learning activity itself. Students with stronger ILM are more likely to engage in deep-level learning and exhibit higher levels of classroom engagement and learning persistence [13]. Although the protective role of ILM is well-established, a critical issue remains: Why do nursing students show significant variability in ILM under identical educational conditions? This variability suggests a dual source of motivation. It stems not only from the immediate educational environment but also from deeper cognitive constructions. Specifically, it is influenced by how students perceive and value their future. Therefore, this gap in knowledge calls for a deeper investigation into the cognitive antecedents that drive ILM, with future time perspective being a promising direction [14].

FTP refers to how individuals cognitively represent and motivationally engage with their future temporal horizons. It enables students to perceive academic tasks as meaningful steps toward achieving their future goals, thereby supporting their needs for competence and autonomy [15]. FTP is a strong predictor of academic achievement and career success in the healthcare professions [16, 17]. Studies have shown that FTP is closely related to learning motivation: higher levels of FTP enhance motivation and reduce academic burnout [18]. In nursing education, FTP positively influences nursing students’ ILM (the mediator) and subsequently reduces covert classroom disengagement (the behavioral outcome) [19, 20]. FTP promotes sustained learning investment through multiple pathways. Specifically, it first cognitively connects current educational tasks to future professional competencies. It further internalizes the value of career-relevant learning. Finally, it strengthens autonomous regulation via alignment with one’s future self [21].

**Fig. 1:**
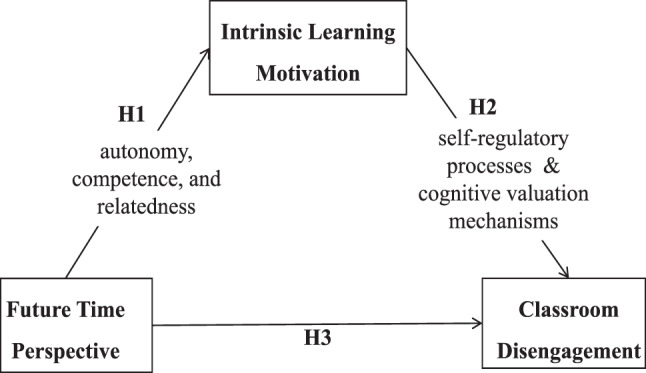
An integrated theoretical model (SDT & SCT & MCIM) of how future time perspective reduces classroom disengagement via intrinsic learning motivation. Note: H: Hypothesis; H1: future time perspective enhances intrinsic learning motivation through the satisfaction of basic psychological needs (autonomy, competence, and relatedness); H2: intrinsic learning motivation reduces classroom disengagement through the mediating mechanisms of self-regulatory processes and cognitive valuation of learning tasks; H3: future time perspective is directly and negatively correlated with classroom disengagement. The model posits intrinsic learning motivation as a central mediator in the cognitive–motivation–behavior pathway

According to Social Cognitive Theory (SCT), nursing students with high FTP perceive classroom disengagement as a threat to their professional self-concept, thereby enhancing their self-regulatory efforts [22]. The Motivation – Cognition Integration Model (MCIM) further proposes that FTP confers immediate value to learning tasks by establishing a goal hierarchy, thereby mitigating the adverse effects of high-stress learning environments [23]. Together, these theories form an integrated theoretical framework. To present this conceptualization, we propose a conceptual model (see Fig. 1). The model delineates a causal pathway wherein FTP, as a cognitive antecedent, enhances ILM by satisfying psychological needs. This enhanced ILM, in turn, reduces classroom disengagement through self-regulatory processes and cognitive valuation mechanisms.

Nevertheless, this cognitive – motivation – behavior mediation pathway remains insufficiently validated, particularly within high-acuity and high-expectation training contexts of nursing education [24]. Given the distinctive nature of nursing education, empirically validating this mediation model is both a theoretical and practical priority [25]. This study represents one of the first large-scale empirical examinations of the integrated future time perspective–intrinsic learning motivation–disengagement pathway in nursing education. Our findings would:

1. Advance theoretical understanding of learning engagement mechanisms in nursing education through an integrated theoretical framework.
2. Provide evidence-based insights for optimizing pedagogical interventions.
3. Inform strategies to mitigate academic disengagement in high-stakes clinical training environments

## Aim

This study has two main aims. First, it investigates the correlations between FTP, ILM, and classroom disengagement among nursing students. Second, it tests the mediating effect of ILM on the relationship between FTP and classroom disengagement. The study thereby provides scientific evidence and practical directions for improving nursing education and reducing classroom disengagement rates.

## Method

### Study design

This study was reported in accordance with the Strengthening the Reporting of Observational Studies in Epidemiology (STROBE) checklist [26] (Appendix 1). The study design adopted a descriptive cross-sectional survey.

### Participants and sample

A total of 889 nursing students from six undergraduate institutions in Shaanxi Province were selected as participants using a convenience sampling method between December 2024 and March 2025. Participants were required to meet the following inclusion criteria: ① being full-time nursing students; ② voluntarily participating in the study and providing informed consent. Exclusion criteria included being unable to participate due to medical or personal leave during the investigation period.

The sample size was calculated based on the method proposed by Kendall, which suggests that the sample size should generally be ten times the number of study variables. The calculation included 21 variables, comprising 9 general demographic items and 12 dimensions from the scale. Considering that 20% of the questionnaires might be invalid, the calculated sample size was *N* = 21 × 10× (1+20%) = 252. A total of 920 questionnaires were distributed in this study, of which 889 were valid, yielding an effective recovery rate of 96.6%.

### Data collection

A cross-sectional survey was conducted among undergraduate nursing students from six public universities in Shaanxi Province between December 2024 and March 2025. Ethical approval was obtained from the administrative departments of the participating institutions. Following a detailed explanation of the study’s purpose and procedures, written informed consent was secured from all participants. With the assistance of head teachers, questionnaires were distributed and collected within a 20-minute timeframe. All returned questionnaires were screened for completeness. Thirty-one questionnaires with incomplete or identical responses were excluded, resulting in 889 valid responses for final analysis (valid response rate: 96.6%).

### Ethical considerations

This study has been approved by the Ethics Committee of Xi’an Medical University (Approval Number: XYLS2025156). In strict adherence to the principles of the Declaration of Helsinki and relevant Chinese regulations, all participants provided written informed consent after being fully informed of the study details. We assured that their involvement in this study would not affect their academic performance, future employment, or relationships with their schools or internship units. Data were stored in encrypted format, accessible only to the research team, and used solely for research purposes. The anonymity and confidentiality were guaranteed as no personally identifiable information was collected. Participants could withdraw from or discontinue the survey at any time without any penalty. As this study involved only questionnaire-based data collection without any interventions or experimental procedures. Its ultimate aims to provide theoretical insights for nursing education and management.

## Measurements

### General characteristics of participants

A self-designed general information questionnaire was used to collect demographic characteristics, including nursing students’ gender, academic performance, residence, mother and father education level, family’s economic status, reason for choosing nursing, student leadership experience, and intention to pursue graduate studies, totaling 9 items.

### Chinese version of the future time perspective scale

The Future Time Perspective Scale was used to assess college students’ FTP. It was developed by Song et al. [27]. The scale consists of five dimensions: future time efficacy, distant goal orientation, behavioral commitment, future imagery, and purpose awareness, totaling 20 items. It uses a 4-point Likert scale ranging from 1 (‘not consistent’) to 4 (‘very consistent’). Total scores range from 20 to 80, with higher scores indicating a greater future time insight. In this study, the Cronbach’s α coefficient was 0.865.

### Chinese version of the intrinsic learning motivation scale

The Chinese version of the Intrinsic Learning Motivation Scale, adapted from the subscale developed by Amabile et al. [28] and translated by Chi et al. [29], was used to assess intrinsic learning motivation. This subscale consists of 14 items across two dimensions: Challenge and Enthusiasm. Items were rated on a 4-point Likert scale ranging from 1 (strongly disagree) to 4 (strongly agree). Total scores range from 14 to 56, with higher scores indicating greater intrinsic learning motivation. In this study, the scale demonstrated good internal consistency, with a Cronbach’s α of 0.801.

### Chinese version of the classroom disengagement scale

The Classroom Disengagement Scale was used to measure classroom disengagement behaviors. It was developed by Su et al. [30]. This scale comprises 28 items across five dimensions: academic cognition, learning habits, self-regulation, classroom perception, and learning environment. All items employ a 5-point Likert scale ranging from 1 (“strongly disagree”) to 5 (“strongly agree”). The total score ranges from 28 to 140, with higher scores indicating more severe classroom disengagement behaviors. In this study, the overall Cronbach’s α coefficient was 0.747.

### Statistical analysis

The IBM SPSS Statistics software version 26.0 (IBM Corp, Armonk, NY, USA) was used to perform data analysis. All tests were two-tailed, and a p-value of less than 0.05 was considered statistically significant. Common method bias was assessed using Harman’s single-factor test, with the criterion that the first unrotated factor should explain less than 40% of the total variance [31, 32]. The normality of continuous variables was assessed using the Kolmogorov-Smirnov test. Normally distributed continuous variables are presented as mean (M) ± standard deviation (SD), while categorical variables are presented as frequency (percentage). Pearson correlation analysis was used to explore the relationships among FTP, ILM, and classroom disengagement. Hierarchical Multiple Regression (HMR) analysis was initially used for two purposes: to identify significant predictors of classroom disengagement among nursing students, and to preliminarily assess ILMs potential mediating role in the FTP-disengagement relationship. To formally test the mediating effect of ILM, the SPSS PROCESS Macro (version 4.0, Model 4; Hayes, 2012) was employed. The significance of the indirect effect was tested using the bias-corrected bootstrap method with 5000 resamples to generate 95% confidence intervals (CI). A significant mediating effect is indicated if the 95% CI does not include zero. The proportion of the total effect mediated by ILM was calculated as the ratio of the indirect effect (ab) to the total effect (c), where c = c′+ ab (c’ is the direct effect).

## Results

### Common method bias test

Harman’s single-factor test revealed that the variance explained by the first component was 21.73%, which did not exceed the 40% threshold, suggesting no serious common method bias [31, 32].

### Demographic characteristics of nursing students

A total of 889 nursing students participated in this study. The cohort was predominantly female (89.5%). In terms of academic performance, most students (51.2%) self-reported as “Average”. A significant majority (66.4%) expressed no intention to pursue postgraduate studies. Geographically, most students (71.7%) originated from rural areas. Regarding family background, 42.2% perceived their family’s economic status as “Moderate”, and the education level of most parents was junior high school (60.8% for mothers, 43.8% for fathers). Half of the students (50.2%) reported that their reason for choosing nursing was due to “My family’s wishes and insistence”, and a majority (60.5%) had student leadership experience. The detailed demographic profile is presented in Table 1.

**Table 1:** Demographic and academic characteristics of the participating nursing students (*N* = 889)

Item	Category	Frequency (n)	Percentage (%)
Gender	Male	93	10.5
	Female	796	89.5
Perceived family economic status	Struggling with basic needs	19	2.1
	Managing essentials only	71	8.0
	Moderate disposable income	375	42.2
	Financially secure	321	36.1
	High net worth	103	11.6
Reason for choosing nursing	I like the nursing profession	363	40.8
	My family’s wishes and insistence	446	50.2
	Not having enough points for another department	33	3.7
	Other reasons	47	5.3
Intention to pursue graduate studies	Yes	299	33.6
	No	590	66.4
Residence	Urban	252	28.3
	Rural	637	71.7
Student leadership experience	Yes	538	60.5
	No	351	39.5
Self-reported academic performance	Very poor	5	0.6
	Below Average	28	3.1
	Average	455	51.2
	Satisfactory	334	37.6
	Excellent	67	7.5
Mother’s education level	Primary school or below	202	22.7
	Junior high school	364	40.9
	Senior high school	239	26.9
	College/University or above	84	9.4
Father’s education level	Primary school or below	132	14.8
	Junior high school	389	43.8
	Senior high school	268	30.1
	College/University or above	100	11.2

Note: This table summarizes the background characteristics of the study sample. These variables were included as control variables in the subsequent regression analysis due to their potential influence on students’ learning engagement and motivation. The categories for ‘perceived family economic status’ and ‘academic performance’ were based on student self-assessment on a 5-point Likert scale

### The scores of future time perspective, intrinsic learning motivation, and classroom disengagement

The descriptive statistics for the key study variables are presented in Table 2. The mean score for Classroom Disengagement was 88.055 ± 12.085. For the positive constructs, the mean scores were 39.095 ± 5.247 for ILM and 53.696 ± 8.610 for FTP.

**Table 2:** Descriptive statistics for the key study variables

Variable	Number of Items	Possible Score Range	Actual Score (M ± SD)
Classroom Disengagement	28	28 - 140	88.055 ± 12.085
Intrinsic Learning Motivation	14	14 - 56	39.095 ± 5.247
Future Time Perspective	20	20 - 80	53.696 ± 8.610

Note: M, Mean; SD, Standard Deviation. The scores represent the sum of all item responses for each scale. Higher scores on the ‘Classroom Disengagement’ scale indicate higher levels of disengagement, while higher scores on ‘Intrinsic Learning Motivation’ and ‘Future Time Perspective’ indicate higher levels of these positive constructs

### The relationship between future time perspective, intrinsic learning motivation, and classroom disengagement

Pearson correlation analysis revealed that classroom disengagement was negatively correlated with FTP (*r* = −0.743, *p* < 0.001) and ILM (*r* = −0.738, *p* < 0.001), while ILM was positively correlated with FTP (*r* = 0.613, *p* < 0.001). See Table 3.

**Table 3:** Correlation analysis of future time perspective, intrinsic learning motivation, and classroom disengagement

Variables	1	2	3
Future Time Perspective	1		
Intrinsic Learning Motivation	0.613**	1	
Classroom Disengagement	−0.743**	−0.738**	1

Note: ** *p* < 0.001; 1. Future Time Perspective; 2. Intrinsic Learning Motivation; 3. Classroom Disengagement

### Hierarchical regression analysis

Hierarchical regression analysis was conducted to investigate the relationships among demographic variables, FTP, ILM, and classroom disengagement.

Step 1 (Control Variables): Demographic factors (gender, academic performance, residence, parental education, graduate study intention, student leadership roles, family economic status, and nursing-major choice reason) explained 8.3% of the variance in classroom disengagement (*R*^*2*^ = 0.083).

Step 2 Adding FTP as a predictor showed a significant negative effect on classroom disengagement (*β* = −0.732, *p* < 0.001), accounting for an additional 47.6% of the variance (*ΔR*^*2*^ = 0.476).

Step 3 With ILM included as a mediator, the effect of FTP remained significant but decreased (*β* = −0.463, *p* < 0.001), and ILM itself was negatively associated with disengagement (*β* = −0.448, *p* < 0.001). This step explained a further 12.3% of the variance (*ΔR*^*2*^ = 0.123), supporting a partial mediation role of ILM between FTP and classroom disengagement. See Table 4.

**Table 4:** Hierarchical regression analysis predicting classroom disengagement (*N* = 889)

Step	Predictors	R^2^	ΔR^2^	β	SE	P
**1**	Demographic variables	0.083		-	-	-
**2**	Future Time Perspective	0.559	0.476	−0.732	0.033	< 0.001
**3**	Future Time Perspective	0.682	0.123	−0.463	0.035	< 0.001
	Intrinsic Learning Motivation			−0.448	0.059	< 0.001

Note: β, standardized regression coefficient; SE, standard error; *R*^*2*^ , coefficient of determination; *ΔR*^*2*^ , change in *R*^*2*^ . Demographic variables included gender, academic performance, residence, parental education, etc. Dashes (-) indicate that the statistics are not applicable for the block of demographic variables as a whole

### Testing of intrinsic learning motivation as a mediator variable

**Fig. 2:**
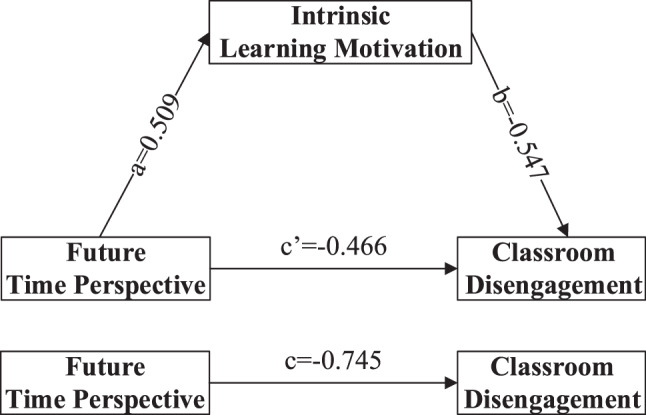
Mediation effect model of intrinsic learning motivation

In this study, Model 4 in the SPSS macro program PROCESS was used to examine the mediating role of ILM between FTP and classroom disengagement. The results showed that FTP significantly predicted ILM (*a* = 0.509, SE = 0.022, *p* < 0.001). When both FTP and ILM were entered into the regression equation simultaneously, FTP remained a significant predictor of classroom disengagement (*c’* = −0.466, SE = 0.024, *p* < 0.001), and ILM also significantly predicted classroom disengagement *(b* = −0.547, SE = 0.029, *p* < 0.001). The bias-corrected percentile Bootstrap analysis indicated a significant mediating effect of ILM (*ab* = −0.279, Boot SE = 0.019, 95% CI: −0.319 to −0.241). The mediation proportion was 37.4% (*ab/(ab + c’*) * 100%), suggesting a partial mediating role. The relationships among the variables are presented in Fig. 2 and Table 5.

**Table 5:** Mediation analysis of future time perspective, intrinsic learning motivation, and classroom disengagement

Path or Effect	Effect Size	SE	Boot LLCI	Boot ULCI
**Direct and Path Effects**				
FTP → ILM (a path)	0.509	0.022	0.466	0.553
ILM → CD (b path)	−0.547	0.029	−0.604	−0.490
FTP → CD (c’ path, direct)	−0.466	0.024	−0.513	−0.419
**Total and Indirect Effects**				
Total effect (c path)	−0.745	0.023	−0.789	−0.700
Indirect effect (a * b)	−0.279	0.019	−0.319	−0.241

Note: FTP, Future Time Perspective; ILM, Intrinsic Learning Motivation; CD, Classroom Disengagement; SE, standard error; Boot LLCI/ULCI, bias-corrected bootstrap 95% lower and upper-level confidence interval (5000 bootstrap samples). All direct effects and the indirect effect are significant as the confidence intervals do not include zero

## Discussion

Grounded in Self-Determination Theory, this study examined the antecedents of nursing students’ classroom disengagement. It confirmed that ILM acts as a partial mediator between FTP and classroom disengagement.

The study findings revealed that Chinese nursing students’ overall classroom disengagement score was 88.055 ± 12.085 (theoretical median = 84), indicating a moderate level of disengagement [33]. This score was significantly higher than those reported in previous studies by Wang et al. [4]. This discrepancy may be attributable to regional variations among study populations, influenced by geographic, economic, and cultural determinants. Deeper analysis suggested that classroom disengagement among nursing students may be a key predictor of deficient learning motivation. This motivational deficit arises from the dual interplay of passive specialty selection (e.g., familial or societal pressures) and acute reality stressors (e.g., high academic workloads coupled with clinical demands). Only 40.8% of participants in our study selected nursing voluntarily. This finding aligns with South Korean data, where merely 41.5% of nursing students chose the profession based on interest and aptitude [34]. These results indicate that nearly 60% of students lack intrinsic professional identity and learning enthusiasm, which represents the fundamental motivational deficit underlying low classroom engagement. Concurrently, 52.3% of participants perceived their household economic status as average or below, suggesting that over half face financial pressures. Such constraints may lead students to prioritize utilitarian factors (e.g., job security, employment stability) over personal interests in career selection. Korean data support this utilitarian orientation. During economic recessions, nursing specialty choices primarily respond to clinical workforce shortages [35]. Such “compulsory selection” stems from socioeconomic survival needs. It suppresses intrinsic professional enthusiasm, compromises self-directed learning initiative, and predisposes students to engagement deficits like classroom disengagement. Overall, two factors show a significant association: a high proportion of involuntary professional choices and widespread economic pressure. Together, they were associated with insufficient learning engagement and moderate classroom disengagement levels. These levels appear higher compared to some similar studies. Educators should foster intrinsic motivation by integrating meaningful, real-world applications into the curriculum. Additionally, providing support to alleviate students’ financial and academic pressures. Encouraging active learning and enhancing career adaptability can further help reduce classroom disengagement behaviors.

Pearson correlation analysis revealed a significant negative correlation between nursing students’ ILM and classroom disengagement (*r* = −0.738, *p* < 0.001). This finding is consistent with previous research [36]. Intrinsic motivation is a core psychological process that drives learning behaviors and directly influences the quality of academic engagement. This is evidenced by Howard et al. [37], who found that nursing students with higher intrinsic motivation demonstrate greater learning interest, initiative to overcome obstacles, and resistance to distractions. This reduces behaviors like classroom distraction and passive avoidance. Self-Determination Theory provides a strong theoretical foundation for this relationship [19]. Nursing students’ intrinsic motivation comes from several sources. These include their inherent curiosity about nursing knowledge, desire to master skills, and strong identification with the professional value of “saving lives” [38]. This intrinsic drive shifts learning behavior from a passive compliance model to a self-regulated approach. These students establish clear self-management goals [39] and maintain high levels of attention in class [40]. This capability allows them to transform pressures, including the notably rapid growth of medical knowledge (doubling every 73 days [41]) and academic demands, into persistent motivation, showcasing robust learning resilience [42]. The unique nature of nursing education further underscores the critical link between intrinsic motivation and classroom disengagement. This is critical because deficits in nursing technical skills directly compromise patient safety [43]; likewise, classroom disengagement can undermine clinical knowledge, jeopardizing future practice [44]. On the other hand, nursing requires continuous adaptation to evolving knowledge [45]. Intrinsic motivation is characterized by its insensitivity to external interference and long-term stability [46]. These characteristics make it a core driver for sustained academic engagement throughout the nursing curriculum. Furthermore, as students internalize nursing values like care ethics and professional responsibility [47], their professional commitment deepens [39]. This process naturally helps to suppress avoidant learning behaviors. To address these issues, educators should incorporate interactive and clinically relevant teaching methods. Providing consistent feedback and cultivating a supportive learning environment also contribute to boosting engagement and intrinsic motivation.

Correlation analysis revealed that higher levels of FTP are associated with lower rates of classroom disengagement (*r* = −0.743, *p* < 0.001). This finding is consistent with existing theoretical literature, which views FTP as a core cognitive motivational resource [23]. Specifically, Husman and Lens [23] argue that FTP reflects an individual’s belief in future goals and its guiding role in current decision-making. For nursing students, a stronger FTP facilitates their understanding of the connection between current learning and long-term career objectives [48]. This allows them to perceive academic tasks as meaningful steps toward achieving future goals. Such cognitive reframing strengthens students’ intrinsic motivation and their ability to engage in rational thinking and self-regulation [49]. As a result, students can anticipate the long-term consequences of their current behaviors and engage in more planned learning [50, 51]. These self-regulatory capacities are vital for maintaining sustained academic engagement.

From a self-identity standpoint, the “future work self” acts as a bridge that connects nursing students’ current learning endeavors with the development of their ideal professional identity. This connection fosters more positive attitudes toward learning tasks and enhances their sense of self-efficacy and control. These cognitive and self-regulatory mechanisms underpin how FTP strengthens intrinsic motivation among nursing students [52]. The resulting enhancement in motivation encourages more active learning participation, thereby effectively reducing classroom disengagement. Consequently, it is recommended that educators highlight the explicit connection between current learning and future career goals through curricular integration and mentorship programs. Furthermore, incorporating goal-setting exercises and reflective practices into teaching strategies may help reinforce these cognitive mechanisms.

This study reveals that ILM partially mediates the relationship between FTP and classroom disengagement among nursing students. This mediation accounts for 37.4% of the total effect. Future time perspective enables students to construct a vivid “future nurse self” [53]. This imagined self acts as a cognitive bridge. It makes the connection between current theoretical learning and future clinical practice psychologically tangible. Through this cognitive bridging mechanism, students perceive their learning tasks as aligned with future professional goals - a form of integrated regulation according to Self-Determination Theory. This alignment fulfills basic psychological needs, particularly autonomy and competence [19, 54]. As these needs are met, intrinsic learning motivation increases. Students would discover an inherent interest in learning when they connect it to their valued future identity.

The generation of motivational energy is actualized via self-regulatory mechanisms [55]. Students with stronger ILM demonstrate improved metacognitive strategies. They proactively plan their studies, monitor their comprehension, and resist disengagement impulses when confronting distractions or challenging materials [22, 50]. This self-regulatory capacity is supported by both Social Cognitive Theory and the Motivation-Cognition Integration Model. It explains the strong negative correlation found between ILM and disengagement (*r* = −0.738, *p* < 0.001). Essentially, FTP provides the purpose (“why”) for learning, while ILM enables the process (“how”) through self-sustained engagement.

The partial mediation effect indicates that other pathways exist alongside intrinsic learning motivation. First, FTP may directly reduce disengagement through opportunity cost awareness. Students with strong future perspectives may perceive disengagement as wasteful of goal-attainment resources, even when they lack immediate interest in the topic [23]. Second, other parallel mediators likely work together with ILM. We propose professional identity and academic emotions as key candidates. FTP could potentially accelerate professional identity development [56]. At the same time, the positive affect from outlook may buffer against negative emotions that lead to disengagement [57]. Future studies should examine an expanded model incorporating professional identity as an additional mediator and academic emotions as moderators.

## Implications

Our study establishes that Future Time Perspective (FTP) enhances intrinsic motivation by strengthening goal connection. Based on this key finding, we propose three intervention strategies for nursing education. These strategies are both theoretically grounded and contextually appropriate. First, to strengthen future self-continuity, we recommend systematically integrating “future self” visualization exercises and narrative workshops into the curriculum. Additionally, establishing a clinical mentorship program would be beneficial. In this program, frontline nurses would share authentic stories about applying theoretical knowledge to clinical decision-making. These activities help build robust emotional and cognitive connections between students’ current learning self and future professional self. Second, for goal hierarchy integration, “goal mapping” workshops can guide students in decomposing long-term career visions. This process involves breaking them into specific academic goals and actionable tasks. To enhance the future value of knowledge modules, it explicitly links theoretical learning (e.g., cardiovascular pharmacology) to clinical responsibilities (e.g., medication administration in cardiac ICU). Third, creating a need-supportive learning environment through SDT principles is essential. This environment would be designed to support autonomy through flexible learning paths and assessment choices. Secondly, it would enhance competence by providing timely formative feedback and well-designed clinical simulations that build mastery. Finally, it would promote relatedness by fostering collaborative learning communities with active peer interaction and supportive faculty engagement. Together, these multifaceted approaches address both motivational and cognitive aspects of learning engagement in nursing education.

## Limitation

Several limitations should be acknowledged. First, the use of convenience sampling from only six universities in Shaanxi Province undermines the external validity and generalizability of the findings. The demographic and regional characteristics of the sample may not fully represent the diverse population of nursing students across China. This potential limitation could restrict the applicability of our results to other contexts with different educational policies and cultural backgrounds. Second, the cross-sectional design precludes causal inferences. Future research would benefit from employing longitudinal designs that could track the development of classroom disengagement over time and establish causal relationships among the variables. Third, the reliance on self-report measures introduces potential biases, such as recall errors and social desirability effects. Subsequent studies should adopt multi-method approaches, including behavioral observations and learning analytics, to triangulate data and enhance objectivity. Furthermore, future research should explore theoretical frameworks beyond intrinsic motivation to achieve a more comprehensive understanding of motivational processes. Finally, intervention studies based on these findings are warranted to translate theoretical insights into practical solutions. Future multi-site or nationwide studies with randomized sampling are needed to enhance the generalizability of these findings and to develop effective evidence-based interventions in nursing education.

## Conclusion

This study demonstrated that intrinsic learning motivation (ILM) partially mediated the relationship between future time perspective (FTP) and classroom disengagement among nursing students, accounting for 37.4% of the total variance. The significant negative association between FTP and classroom disengagement underscores the role of ILM as a key explanatory mechanism. These findings highlight future-oriented thinking as a pivotal target for educational interventions designed to bolster intrinsic motivation and mitigate disengagement. Despite these contributions, the cross-sectional design of this study precludes definitive causal inferences, and the use of convenience sampling may limit the generalizability of the results. Future research should employ longitudinal or intervention designs to verify causal relationships and assess the effectiveness of strategies developed from these constructs.

## Supplementary Information

Below is the link to the electronic supplementary material.

Supplementary Material 1

## Data Availability

The datasets used and/or analyzed during the current study are available from the first author and the corresponding author on reasonable request.
