# Supplementary material for: Mediating role of intrinsic learning motivation in the relationship between future time perspective and classroom disengagement among Chinese nursing students: a cross-sectional study
Source: BMC Nurs. 2026 Jan 8;25:126. doi: 10.1186/s12912-025-04280-6 (PMC12882286; doi:10.1186/s12912-025-04280-6)
Supplement: Supplementary file 1 — Supplementary Material 1 [file 12912_2025_4280_MOESM1_ESM.pdf]

Appendixd 1 The STROBE Statement-checklist of items that should be addressed in reports of observational studies.

	Item No.	Recommendation
**Title and Abstract**	1	(a) Indicate the study's design with a commonly used term in the title or the abstract
		(b) Provide in the abstract an informative and balanced summary of what was done and what was found
**Introduction**		
Background/rationale	2	Explain the scientific background and rationale for the investigation being reported
Objectives	3	State specific objectives, including any prespecified hypotheses
**Methods**		
Study design	4	Present key elements of study design early in the paper
Setting	5	Describe the setting, locations, and relevant dates, including periods of recruitment, exposure, follow-up, and data collection
Participants	6	(a) Cohort study– Give the eligibility criteria, and the sources and methods of selection of participants. Describe methods of follow-up<br>Case-control study– Give the eligibility criteria, and the sources and methods of case ascertainment and control selection. Give the rationale for the choice of cases and controls<br>Cross-sectional study– Give the eligibility criteria, and the sources and methods of selection of participants<br>(b)Cohort study– For matched studies, give matching criteria and number of exposed and unexposed<br>Case-control study– For matched studies, give matching criteria and number of controls per case
Variables	7	Clearly define all outcomes, exposures, predictors, potential confounders, and effect modifiers. Give diagnostic criteria, if applicable

Data sources/ measurement	8[a]	For each variable of interest, give sources of data and details of methods of assessment (measurement). Describe comparability of assessment methods if there is more than one group
Bias	9	Describe any efforts to address potential sources of bias
Study size	10	Explain how the study size was arrived at
Quantitative variables	11	Explain how quantitative variables were handled in the analyses. If applicable, describe which groupings were chosen and why
Statistical methods	12	(a) Describe all statistical methods, including those used to control for confounding<br>(b) Describe any methods used to examine subgroups and interactions<br>(c) Explain how missing data were addressed<br>(d)Cohort study– If applicable, explain how loss to follow-up was addressed<br>Case-control study– If applicable, explain how matching of cases and controls was addressed<br>Cross-sectional study– If applicable, describe analytical methods taking account of sampling strategy<br>(e) Describe any sensitivity analyses
**Results**		
Participants	13[a]	(a) Report the numbers of individuals at each stage of the study (e.g., numbers potentially eligible, examined for eligibility, confirmed eligible, included in the study, completing follow-up, and analysed)<br>(b) Give reasons for non-participation at each stage<br>(c) Consider use of a flow diagram
Descriptive	14[a]	(a) Give characteristics of study participants (e.g., demographic, clinical, social) and information on exposures and potential confounders

		(b) Indicate the number of participants with missing data for each variable of interest
		(c)Cohort study– Summarise follow-up time (e.g., average and total amount)
Outcome data	15[a]	Cohort study– Report numbers of outcome events or summary measures over time<br>Case-control study– Report numbers in each exposure category, or summary measures of exposure<br>Cross-sectional study– Report numbers of outcome events or summary measures
Main results	16	(a) Give unadjusted estimates and, if applicable, confounder-adjusted estimates and their precision (e.g., 95% confidence interval). Make clear which confounders were adjusted for and why they were included<br>(b) Report category boundaries when continuous variables were categorized<br>(c) If relevant, consider translating estimates of relative risk into absolute risk for a meaningful time period
Other analyses	17	Report other analyses done (e.g., analyses of subgroups and interactions, and sensitivity analyses)
**Discussion**		
Key results	18	Summarise key results with reference to study objectives
Limitations	19	Discuss limitations of the study, taking into account sources of potential bias or imprecision. Discuss both direction and magnitude of any potential bias
Interpretation	20	Give a cautious overall interpretation of results considering objectives, limitations, multiplicity of analyses, results from similar studies, and other relevant evidence
Generalisability	21	Discuss the generalisability (external validity) of the study results
Other information		
Funding	22	Give the source of funding and the role of the funders for the present study and, if applicable, for the original study on which the present article is based

[a]:Give such information separately for cases and controls in case–control studies, and, if applicable, for exposed and unexposed groups in cohort and

cross-sectional studies.

Note: An Explanation and Elaboration article discusses each checklist item. The STROBE website (http://www.strobe-statement.org/) offers separate checklists for cohort, case–control, and cross-sectional studies.
